# Taking Doctor’s Orders

**Published:** 1917-02

**Authors:** 


### Taking Doctor's Orders.

A country doctor, walking out one day with a Highlander, who boasted he was a staunch teetotaler, resolved to put him to the test. Passing a hotel, he asked him in and ordered two glasses of whisky. After they had "shifted" these and two or three more at the doctor's expense, his friend began to get a wee bit "glib o' the gab." The doctor then, feeling he had him, bluntly asked:

"How does this square wi' your teetotal pretentions?"

"Weel," quoth John, with a quiet smile, "though I'm a stanch teetotaler, I'd be a fule to refuse what the doctor orders."—*Pittsburg Chronicle Telegraph.*

